# Early postnatal development of pyramidal neurons across layers of the mouse medial prefrontal cortex

**DOI:** 10.1038/s41598-019-41661-9

**Published:** 2019-03-25

**Authors:** Tim Kroon, Eline van Hugte, Lola van Linge, Huibert D. Mansvelder, Rhiannon M. Meredith

**Affiliations:** 1 Department of Integrative Neurophysiology, Center for Neurogenomics & Cognitive Research, Vrije Universiteit Amsterdam, De Boelelaan 1085, 1081 HV Amsterdam, The Netherlands; 2 Present Address: MRC Centre for Developmental Neurobiology, Institute of Psychiatry, Psychology & Neuroscience, King’s College London, New Hunt’s House, Guy’s Campus, London, SE1 1UL UK; 3 Present Address: Department Cognitive Neurosciences, Department of Human Genetics, Donders Institute for Brain, Cognition and Behaviour, Radboud University Medical Centre, Geert Grooteplein 10 Noord, 6500 HB Nijmegen, The Netherlands; 4 Present Address: Department of Functional Genomics, Center for Neurogenomics & Cognitive Research, Vrije Universiteit Amsterdam, De Boelelaan 1085, 1081 HV Amsterdam, The Netherlands

## Abstract

Mammalian neocortex is a highly layered structure. Each layer is populated by distinct subtypes of principal cells that are born at different times during development. While the differences between principal cells across layers have been extensively studied, it is not known how the developmental profiles of neurons in different layers compare. Here, we provide a detailed morphological and functional characterisation of pyramidal neurons in mouse mPFC during the first postnatal month, corresponding to known critical periods for synapse and neuron formation in mouse sensory neocortex. Our data demonstrate similar maturation profiles of dendritic morphology and intrinsic properties of pyramidal neurons in both deep and superficial layers. In contrast, the balance of synaptic excitation and inhibition differs in a layer-specific pattern from one to four postnatal weeks of age. Our characterisation of the early development and maturation of pyramidal neurons in mouse mPFC not only demonstrates a comparable time course of postnatal maturation to that in other neocortical circuits, but also implies that consideration of layer- and time-specific changes in pyramidal neurons may be relevant for studies in mouse models of neuropsychiatric and neurodevelopmental disorders.

## Introduction

Pyramidal neurons (PNs) in different cortical layers differ in their expression of molecular markers^1–3^, responses to sensory stimuli^4^, patterns of synaptic connectivity^5^, and morphological properties^6–9^. The neocortex develops in an inside-out manner, with principal cells in superficial layers migrating past earlier-born neurons in deeper layers^10^. Thus, deeper layer neurons reach their destination within the cortex several days earlier than neurons in superficial layer. However, little is known about how neuronal maturation during early stages of development compares between cortical layers. Laminar-specific development of axonal arborisation has been described in mouse barrel cortex^11^, and efforts have been made to determine the development of axonal innervation of different cortical layers, particularly with respect to thalamocortical innervation^12^. It has also been shown that the development of synapses occurs simultaneously across all layers of the cortex^13^. However, because apical dendrites often traverse multiple layers, it is difficult to determine from these data which neurons are targeted by these axons and synapses. Furthermore, we know very little about laminar differences in the development of intrinsic neuronal characteristics like dendritic morphology and membrane properties, because the majority of studies investigating layer specificity have focused on neuronal differences at one particular stage of cortical development^14,15^. In addition, most studies that do assess developmental aspects tend to focus only a single cortical layer^16–18^.

Knowledge of multi-layer neuronal development is essential for understanding the formation and refinement of cortical circuits^19^, as synaptic activity during development is critical to proper circuit wiring^20^, and synaptic impairments during development result in circuit dysfunction. Although studies of neurodevelopmental disorders (NDDs) rarely assess phenotypes at different time points, it is increasingly recognised that NDDs demonstrate age-restricted morphological and/or functional aberrations during sensitive periods of early brain development^21–23^. Whilst many NDD studies now assess developmental trajectories, revealing transient age-specific phenotypes, these studies are often limited to a single type of connection or cortical layer^24–26^. However, previous results have shown that sensitive periods in the cortex can have laminar differences in timing and duration^27^. Hence, studying layer-specific neuronal development may provide new insights into mechanisms underlying NDDs.

The medial prefrontal cortex (mPFC) is involved in several cognitive and executive function processes such as attention and decision-making^28^ and is affected in many NDDs^29^. The mPFC in rodents is exceptional in that it lacks a granular layer 4, which in sensory areas is the main target of thalamic input. A thorough characterisation of rat mPFC pyramidal neurons in young adulthood reveals a diversity of subtypes across deep and superficial layers^6^. A detailed understanding of the development of neurons across mPFC layers would give better insight into the functional maturation of the circuitry in this cortical region and its involvement in NDDs.

To this end, we provide an extensive analysis of the development of dendritic morphology and intrinsic membrane properties of pyramidal neurons in layers 3 and 5 of the mouse mPFC, as well as their synaptic input. We focus on the first postnatal month, as this is a time of rapid development in the rodent cortex^30,31^. We show that morphology and intrinsic membrane properties develop largely simultaneously in both layers. Excitatory inputs onto layer 3 PNs increase rapidly during the second postnatal week, while those onto layer 5 increase more slowly. Inhibitory inputs, on the other hand, develop more slowly in layer 3 than layer 5. This leads to a dynamic ratio of excitation and inhibition that is increased in layer 3 relative to layer 5 at two weeks postnatal. Thus, development of synaptic input follows a markedly different time course in either layer. We suggest that this could result in unique sensitive time windows for synaptic maturation of individual cortical layers in the mouse mPFC.

## Results

### Concurrent development of dendritic morphology across cortical layers

**Figure 1:**
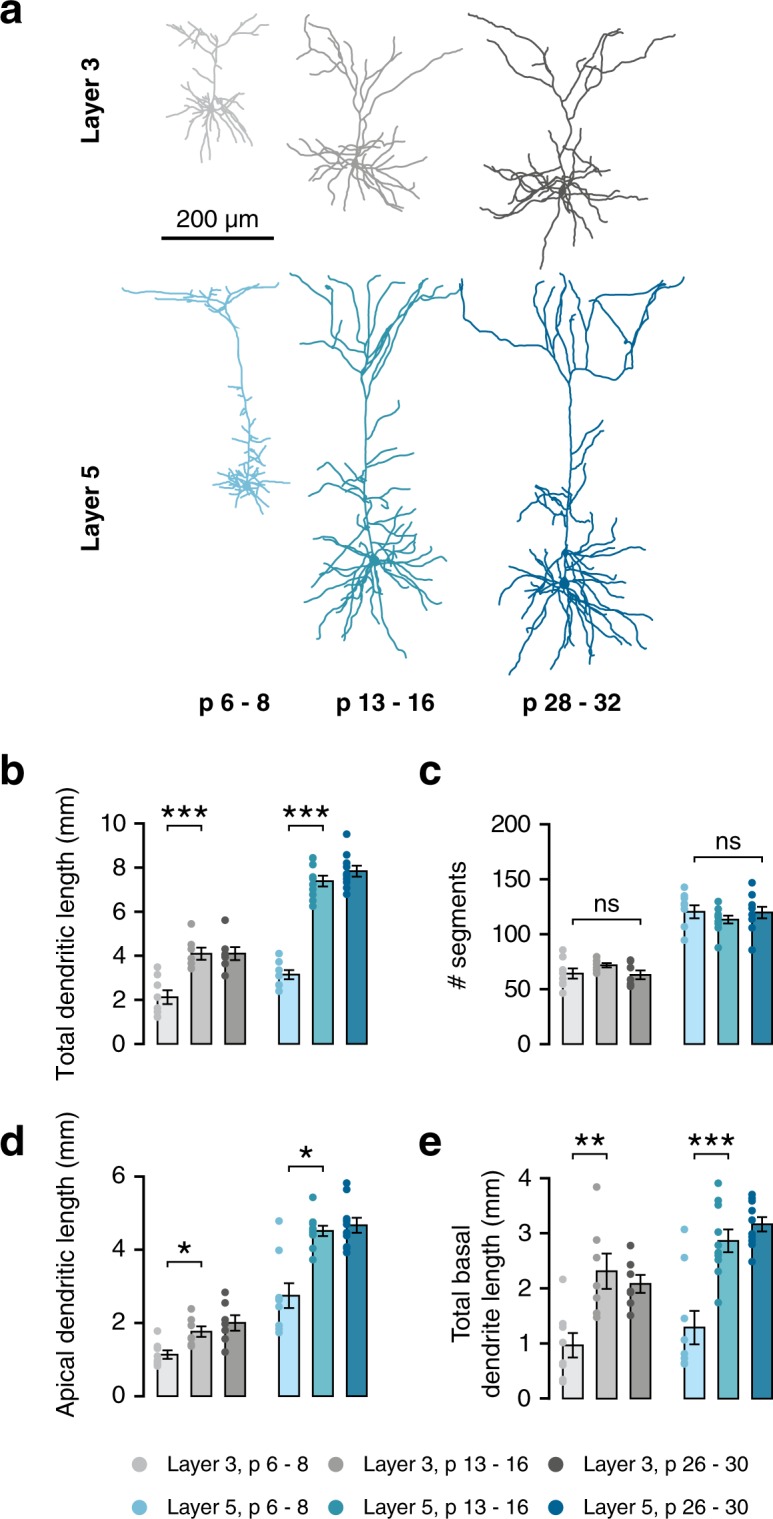
Pyramidal cells in mPFC undergo rapid growth during the second postnatal week. (**a**) Example morphologies of L3 and L5 pyramidal cells from all three age groups. (**b**) Total dendritic length increases between week 1 and week 2 for cells from both layer 3 (F(2,19) = 15.34, p < 0.001; post-hoc w1-w2, p < 0.001) and layer 5 (F(2,24) = 93.65, p < 0.001; post-hoc w1-w2, p < 0.001). (**c**) There is no difference in the overall number of dendritic segments between weeks 1 and 4 (L3, χ^2^(2) = 4.75, p = 0.093; L5, χ^2^(2) = 2.52, p = 0.283). (**d**) Total apical dendritic length increases between weeks 1 and 2 in both layers (L3, F(2,19) = 8.26, p = 0.003; post-hoc w1-w2, p = 0.028; L5, H(2) = 11.66, p = 0.003; post-hoc w1-w2, p = 0.016). (**e**) Total length of all basal dendrites combined increases between 1 and 2 weeks in both layers (L3, F(2,19) = 9.16, p = 0.002; post-hoc w1-w2, p = 0.002; L5, F(2,26) = 20.36, p < 0.001; post-hoc w1-w2, p < 0.001).

For the characterisation of dendritic morphology, 51 neurons were reconstructed from 18 C57Bl/6 mice divided into three age groups: week 1 (w1; postnatal day (P) 6–8), week 2 (w2; P13–16) and week 4 (w4; p26–30). Cells were patched in layer 3 and layer 5 at each age group. Within each cortical layer, there are several subtypes of pyramidal neurons^6^. We selected L5 pyramidal neurons for their large soma size. Consequently, L5 groups only contained broad-tufted cells. For the L3 groups, some slim-tufted neurons were reconstructed, but these were excluded from the final analysis. Figure 1a shows example morphologies from each of the groups.

**Figure 2:**
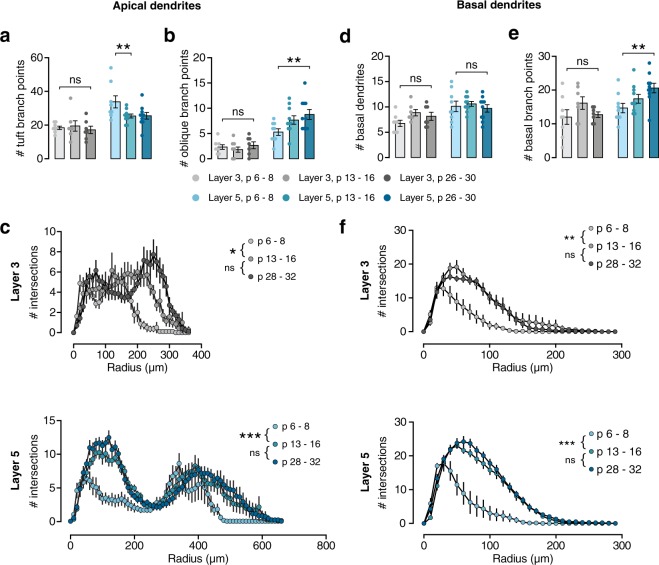
Development of dendritic morphology shows small cell type-specific differences. (**a**) The number of tuft segments does not change in layer 3 (χ^2^(2) = 0.99, p = 0.609). However, number of segments decreases between week 1 and week 2 in neurons of L5, χ^2^(2) = 14.09, p < 0.001, post-hoc w1-w2, p = 0.004). (**b**) The number of branch points in oblique dendrites increases during development of layer 5 cells (χ^2^(2) = 8.36, p = 0.015, post-hoc w1 vs w4, p = 0.013), but not in layer 3 cells (χ^2^(2) = 1.12, p = 0.572). (**c**) Sholl analysis of apical dendrites. In both layers, the pattern of intersections is different between week 1 and 2, but not between week 2 and 4 (L3, F(2,21) = 9.35, p = 0.001; post-hoc w1-w2, p = 0.011; L5, F(2,27) = 15.05, p < 0.001; post-hoc w1-w2, p < 0.001). (**d**) The number of basal dendrites does not change between 1 and 4 weeks in either layer (L3, χ^2^(2) = 2.20, p = 0.333; L5, χ^2^(2) = 0.40, p = 0.819). (**e**) The number of basal branch points is similar between ages in layer 3 cells (χ^2^(2) = 5.22, p = 0.074), but increases in layer 5 between 1 and 4 weeks (χ^2^(2) = 9.43, p = 0.009; post-hoc w1 vs w4, p = 0.006). (**f**) Sholl analysis of basal dendrites. In both layers, the pattern of intersections is different between week 1 and 2, but not between week 2 and 4 (L3, F(2,19) = 8.77, p = 0.002; post-hoc w1-w2, p = 0.003; L5, F(2,27) = 24.65, p < 0.001; post-hoc w1-w2, p < 0.001).

The development of gross morphology occurred concurrently in PNs from both layers. Total dendritic length increased rapidly during the second postnatal week (Fig. 1b; L3, +93%, p < 0.001; L5, +135%, p < 0.001). No significant further growth was seen between weeks 2 and 4 in either layer. The number of dendritic segments per cell did not change during this time in either layer (Fig. 1c), indicating that the overall structure of the cell is formed before the end of the first postnatal week. This same pattern of growth was seen in both apical (Fig. 1d; L3: +54%, p = 0.003; L5: +71%, p = 0.003) and basal dendrites (Fig. 1e; L3, +139%, p = 0.002; L5, + 122%, p < 0.001). Within the apical dendrite, the same pattern was seen in both oblique dendrites and the apical tuft (Table 1). However, more subtle changes in dendritic structure take place from the second postnatal week onward. Layer 5 dendritic tufts exhibited a reduction in the number of segments (Fig. 2a; L5, p < 0.001), showing that some processes are pruned during the second postnatal week. In contrast, the complexity of oblique dendrites increased between weeks 1 and 4, as the number of branch points in oblique dendrites increased (Fig. 2b), which is also reflected in the Sholl analysis of apical dendrites (Fig. 2c, bottom panel). These changes were not observed in L3 PNs, reflecting the differences in morphology between the layers, as both the apical tuft and oblique dendrites are more elaborate in L5 PNs. Although the number of basal dendrites does not change after the first postnatal week (Fig. 2d), the number of basal branch points slowly increases between 1 and 4 weeks only in L5 PNs (Fig. 2e). This is reflected in the Sholl analysis (Fig. 2f), along with the rapid growth of basal dendrites during the second postnatal week. Thus, while some subtype-specific aspects of dendritic development may differ slightly, development of dendritic morphology overall occurs largely in parallel across layers 3 and 5.

**Table 1:** Morphological properties of layer 3 and 5 pyramidal neurons at 1, 2, and 4 weeks. Ages are given as mean ± sd in days postnatal.

*Variable*	Week 1 p 6.9 ± 1.0 n = 8/5	Week 2 p 14.0 ± 0.0 n = 7/5	Week 4 p 28.4 ± 0.8 n = 7/5	*Omnibus test result*
**Layer 3**				
**Total dendritic length (µm)**	***2120***.***1*** ± ***313***.3***	4090.6 ± 275.1	4098.3 ± 293.5	F(2,19) = 15.34 p < 0.001
# dendritic segments	64.6 ± 4.6	72.1 ± 1.9	63.4 ± 4.0	χ ^2^(2) = 4.75 p = 0.093
**Total basal dendritic length (µm)**	***967***.***4*** ± ***221***.8**	2311.8 ± 319.9	2082.6 ± 163.3	F(2,19) = 9.16 p = 0.002
# basal dendrites	6.8 ± 0.6	8.9 ± 0.6	8.1 ± 0.8	χ ^2^(2) = 2.20 p = 0.333
# basal branch points	12.0 ± 2.2	16.1 ± 1.8	12.7 ± 0.8	χ ^2^(2) = 5.22 p = 0.074
**Total apical dendritic length (µm)**	***1152***.***7*** ± ***114***.6*	1777.6 ± 143.8	2015.7 ± 210.7	F(2,19) = 8.26 p = 0.003
# oblique dendrites	5.0 ± 0.8	3.4 ± 0.8	3.3 ± 0.6	χ ^2^(2) = 3.45 p = 0.179
**Total oblique dendrite length (µm)**	*332*.*74* ± *40*.75*	496.75 ± 73.35	616.74 ± 99.02	F(2,18) = 4.13 p = 0.034
# oblique branch points	2.4 ± 0.5	1.9 ± 0.5	2.7 ± 0.7	χ ^2^(2) = 1.12 p = 0.572
**Total apical tuft dendritic length(µm)**	***735***.***7*** ± ***117***.2*	1,252.1 ± 113.8	1,290.6 ± 116.4	F(2,19) = 7.40 p = 0.004
# apical tuft segments	18.5 ± 1.0	19.6 ± 3.1	17.3 ± 2.2	χ ^2^(2) = 0.99 p = 0.609
**Layer 5**				
***Variable***	**Week 1 p 6**.**9** ± **0**.**9 n** **=** **9/5**	**Week 2 p 14**.**0** ± **0**.**0 n** **=** **10/5**	**Week 4 p 28**.**9** ± **0**.**9 n** **=** **10/5**	***Omnibus test result***
**Total dendritic length (µm)**	***3142***.***3*** ± ***233***.5***	7383.3 ± 245.9	7838.0 ± 250.9	F(2,24) = 93.65 p < 0.001
# dendritic segments	120.7 ± 5.8	113.6 ± 3.5	120.0 ± 5.2	χ ^2^(2) = 2.52 p = 0.284
**Total basal dendritic length (µm)**	***1288***.***8*** ± ***304***.4***	2862.7 ± 207.6	3164.3 ± 130.3	F(2,26) = 20.36 p < 0.001
# basal dendrites	10.1 ± 1.0	10.6 ± 0.5	9.7 ± 0.7	χ ^2^(2) = 0.40 p = 0.819
**# basal branch points**	*14*.*7* ± *1*.4**	17.4 ± 1.3	20.6 ± 1.4	χ ^2^(2) = 8.36 p = 0.015
**Total apical dendritic length (µm)**	***2756***.***1*** ± ***338***.3*	4519.0 140.9	4673.7 203.3	H(2) = 11.66 p = 0.003
# oblique dendrites	16.3 ± 1.2	13.2 ± 0.6	12.7 ± 0.7	χ ^2^(2) = 5.14 p = 0.077
**Total oblique dendrite length (µm)**	***1001***.***5*** ± ***203***.2*	2143.1 ± 110.8	2316.2 ± 95.0	H(2) = 15.12 p = 0.001
**# oblique branch points**	*5*.*3* ± *0*.6*	7.7 ± 0.8	8.8 ± 1.0	χ ^2^(2) = 8.36 p = 0.015
**Total apical tuft dendritic length(µm)**	***1487***.***8*** ± ***166***.7**	2106.5 ± 70.5	2049.9 ± 146.5	F(2,26) = 6.47 p = 0.005
**# apical tuft segments**	***33***.***9*** ± ***3***.8**	25.5 ± 1.2	25.6 ± 2.0	χ ^2^(2) = 14.09 p < 0.001

N given as number of cells/number of mice. Values are given as mean ± SEM. Asterisks denote post-hoc significance: *p < 0.05, **p < 0.01, ***p < 0.001. Bold values denote statistically significant differences compared to week 2 (in week 1), italics denote statistically significant differences compared to week 4 (in week 1 and 2).

### Concurrent development of electrophysiological membrane properties

**Figure 3:**
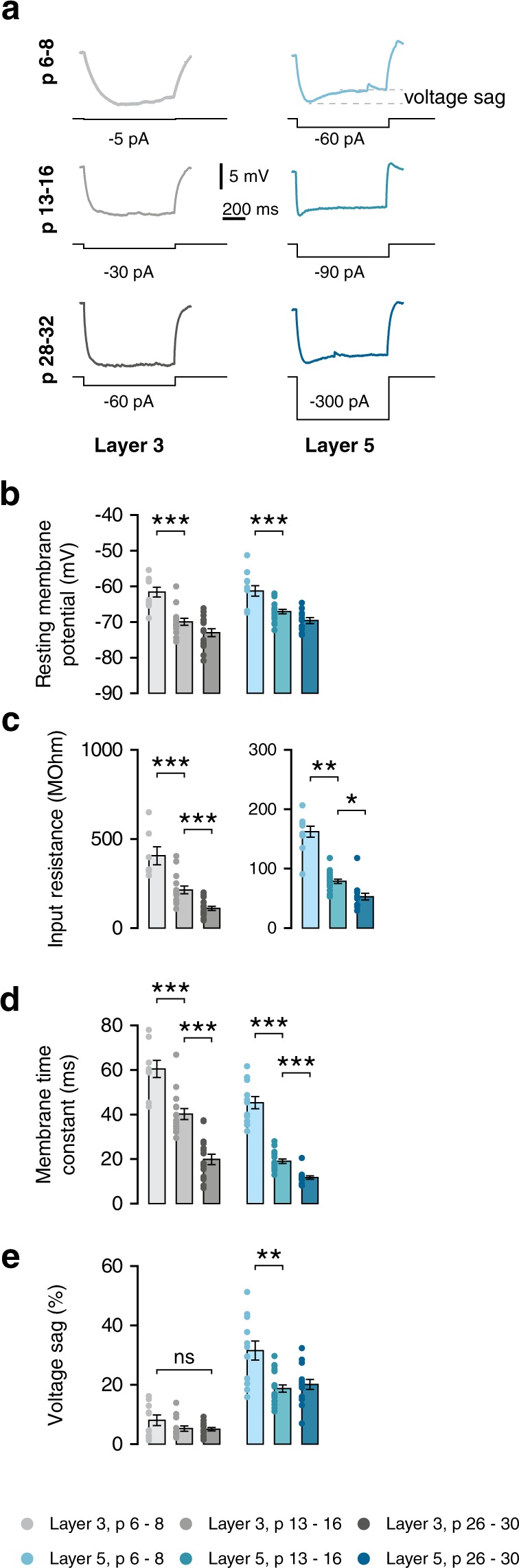
Rapid development of intrinsic membrane properties is similar between layers. (**a**) Example voltage traces in response to negative current injections show the presence of the H-current as early as p6 in layer 5 cells. (**b**) Resting membrane potential becomes more hyperpolarised during the second postnatal week in both layers (L3, F(2,38) = 23.42, p < 0.001; post-hoc w1-w2, p < 0.001; L5, F(2,43) = 18.39, p < 0.001; post-hoc w1-w2, p < 0.001). (**c**) Input resistance decreases strongly during the second postnatal week in both layers, and decreases further until week 4 (L3, F(2,10) = 14.09, p = 0.001; post-hoc w1-w2, p < 0.001; w2 vs w4, p < 0.001; L5, H(2) = 29.33, p < 0.001; post-hoc w1-w2, p = 0.006; w2 vs w4, p = 0.011). (**d**) Membrane time constant of cells of both layers decreases between weeks 1 and 2, and further decreases between weeks 2 and 4 (L3, F(2,36) = 45.42, p < 0.001; post-hoc w1-w2, p < 0.001; w2 vs w4, p < 0.001; L5, F(2,12) = 50.11, p < 0.001; post-hoc w1-w2, p < 0.001; w2 vs w4, p < 0.001). (**e**) There is no prominent voltage sag in layer 3 neurons. Layer 5 neurons do exhibit a voltage sag, which is decreased during the second postnatal week (L5, Welch’s F(2,22) = 6.72, p = 0.005; post-hoc w1-w2, p = 0.006).

**Figure 4:**
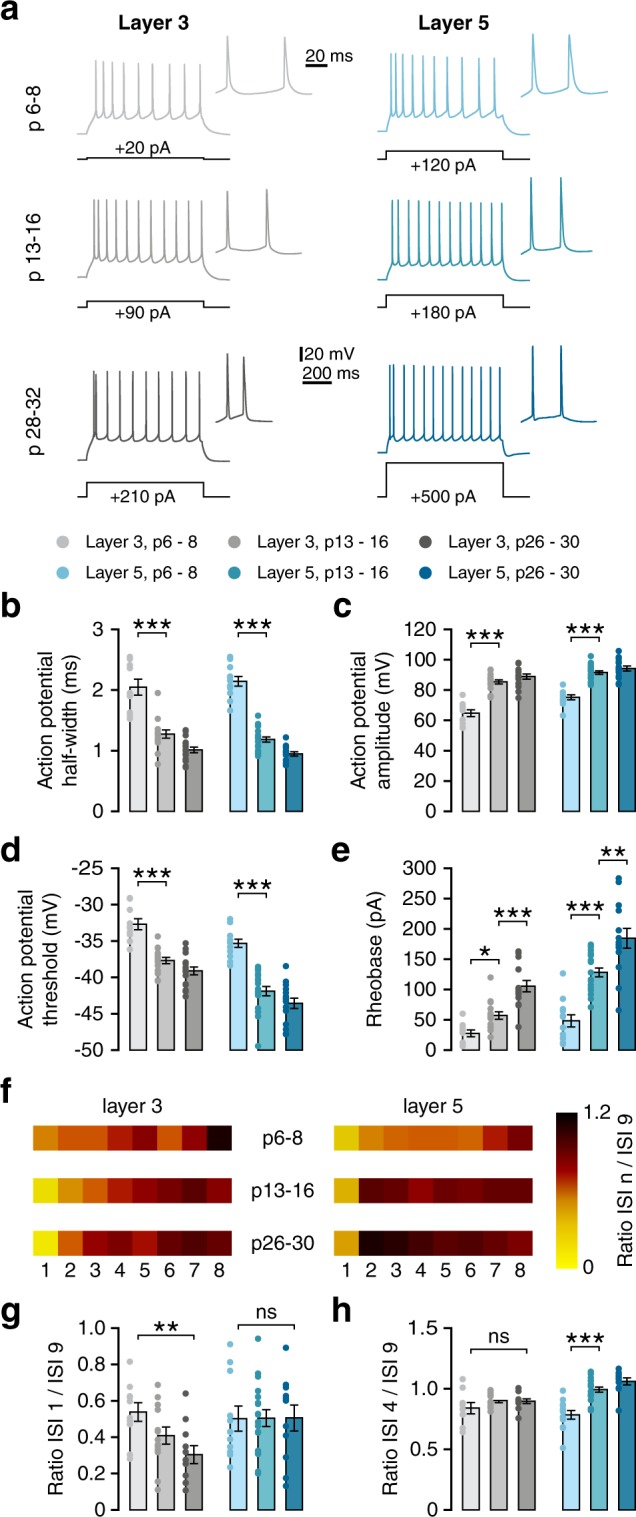
Firing properties in both cortical layers develop largely in parallel. (**a**) Example voltage traces in response to suprathreshold depolarising current step that elicits 10 or more action potentials. Inset: first two action potentials of the same voltage trace. Black lines indicate the amplitude of the current step that elicited the voltage response. (**b**) Action potential halfwidth decreases rapidly during the second postnatal week, decreasing even further afterwards (L3, Welch’s F(2,18) = 25.61, p < 0.001; post-hoc w1-w2, p = 0.001; w2 vs w4, p = 0.011; L5, F(2,42) = 124.67, p < 0.001; post-hoc w1-w2, p < 0.001; w2 vs w4, p = 0.003). (**c**) Action potential amplitude increases during the second postnatal week in neurons of both layers (L3, F(2,37) = 46.85, p < 0.001; post-hoc w1-w2, p < 0.001; L5, F(2,43) = 43.78, p < 0.001; post-hoc w1-w2, p < 0.001). (**d**) Action potential threshold becomes more hyperpolarised between weeks 1 and 2 (L3, F(2,38) = 31.36, p < 0.001; post-hoc w1-w2, p < 0.001; L5, F(2,43) = 36.87, p < 0.001; post-hoc w1-w2, p < 0.001). (**e**) Rheobase increases during development of neurons in both layers (L3, F(2,35) = 23.13, p < 0.001; post-hoc w1-w2, p = 0.036; w2 vs w4, p < 0.001; L5, F(2,40) = 29.74, p < 0.001; post-hoc w1-w2, p < 0.001; w2 vs w4, p = 0.003). (**f**) Spike frequency adaptation represented through ISI ratios. Heatmap colours represent the ratio between the 9^th^ ISI and each of the 8 previous ISIs (numbered below). (**g**) ISI1/9 ratio decreases during development in L3 but not L5 neurons (L3, F(2,31) = 5.21, p = 0.011; post-hoc w1-w4, p = 0.008). (**h**) ISI4/9 ratio increases during development in L5 neurons (F(2,38) = 23.86, p < 0.001, post-hoc w1-w2, p < 0.001).

Postnatal development has a significant effect upon electrophysiological properties of cortical pyramidal neurons, correlating with specific morphological changes during the same period (Zhang 2004). Intrinsic membrane properties were measured from 87 cells from 19 mice, from layers 3 and 5, divided into three age groups: week 1 (w1; postnatal day (P) 6–8), week 2 (w2; P13–16) and week 4 (w4; p26–30) (Figs 3a,4a, Table 2).

**Table 2:** Electrophysiological properties of layer 3 and 5 pyramidal neurons at 1, 2, and 4 weeks. Ages are given as mean ± sd in days postnatal.

*Variable*	Week 1 p 7.0 ± 0.9 n = 10/5	Week 2 p 14.0 ± 0.0 n = 15/5	Week 4 p 28.6 ± 1.0 n = 16/7	*Omnibus test result*
**Layer 3**				
**Resting membrane potential (mV)**	***−61***.***63*** ± ***1***.39***	−69.96 ± 0.98	−73.02 ± 1.09	F(2,38) = 23.42 p < 0.001
**Input resistance (MΩ)**	***405***.***85*** ± ***50***.54***	*213*.*42* ± *21*.95***	110.28 ± 11.54	F(2,10) = 14.09 p = 0.001
**Membrane time constant (ms)**	***0***.***47*** ± ***4***.32***	*40*.*23* ± *2*.48***	19.87 ± 2.34	F(2,36) = 45.42 p < 0.001
**Action potential threshold (mV)**	***−32***.***69*** ± ***0***.75***	−37.68 ± 0.44	−39.11 ± 0.54	F(2,38) = 31.36 p < 0.001
**Action potential amplitude (mV)**	***64***.***76*** ± ***2***.26***	85.41 ± 1.37	88.91 ± 1.78	F(2,37) = 46.85 p < 0.001
**Halfwidth (ms)**	***2***.***05*** ± ***0***.14**	*1*.*27* ± *0*.07*	1.01 ± 0.05	W’s F(2,18) = 25.61 p < 0.001
**Rheobase (pA)**	***27***.***56*** ± ***5***.55*	*57*.*10* ± *6*.17***	105.47 ± 10.32	F(2,35) = 23.13 p < 0.001
**ISI 1**/**ISI 9**	*0*.*58* ± *0*.09*	0.41 ± 0.05	0.30 ± 0.05	F(2,31) = 5.21 p = 0.011
ISI 4/ISI 9	0.84 ± 0.05	0.90 ± 0.02	0.90 ± 0.02	F(2,30) = 1.42 p = 0.258
Sag (%)	8.08 ± 1.78	5.27 ± 0.91	5.05 ± 0.59	W’s F(2,18) = 1.26 p = 0.307
**Layer 5**				
***Variable***	**Week 1 p 7**.**1** ± **0**.**9 n** = **12/5**	**Week 2 p 14**.**0** ± **0**.**0 n** = **19/6**	**Week 4 p 28**.**7** ± **1**.**0 n** = **15/8**	***Omnibus test result***
**Resting membrane potential (mV)**	***−61***.***30*** ± ***1***.45***	−67.08 ± 0.63	−69.62 ± 0.83	F(2,43) = 18.39 p < 0.001
**Input resistance (MΩ)**	***161***.***83*** ± ***10***.06**	*78*.*33* ± *3*.68*	52.60 ± 5.73	H(2) = 29.33 p < 0.001
**Membrane time constant (ms)**	***45***.***33*** ± ***2***.88***	*19*.*01* ± *0*.99***	11.73 ± 0.80	F(2,12) = 50.11 p < 0.001
**Action potential threshold (mV)**	***−35***.***32*** ± ***0***.56***	−41.89 ± 0.64	−43.57 ± 0.71	F(2,43) = 36.87 p < 0.001
**Action potential amplitude (mV)**	***75***.***24*** ± ***1***.62***	91.54 ± 1.17	94.22 ± 1.61	F(2,43) = 43.78 p < 0.001
**Halfwidth (ms)**	***2***.***14*** ± ***0***.08***	*1*.*18* ± *0*.04**	0.95 ± 0.03, n = 15)	F(2,42) = 124.67 p < 0.001
**Rheobase (pA)**	***48***.***27*** ± ***10***.01***	*128*.*15* ± *7*.57**	184.47 ± 17.48	F(2,40) = 29.74 p < 0.001
ISI 1/ISI 9	0.50 ± 0.07	0.50 ± 0.05	0.51 ± 0.07	F(2,37) = 0.001 p = 0.999
**ISI 4**/**ISI 9**	***0***.***79*** ± ***0***.04***	1.03 ± 0.04	1.06 ± 0.03	F(2,38) = 23.86 p < 0.001
**Sag (%)**	***31***.***52*** ± ***3***.20**	18.76 ± 1.23	20.13 ± 1.72	W’s F(2,22) = 6.72 p = 0.005

N given as number of cells/number of mice. Values are given as mean ± SEM. W’s F = Welch’s F. Asterisks denote post-hoc significance: *p < 0.05, **p < 0.01, ***p < 0.001. Bold values denote statistically significant differences compared to week 2 (in week 1), italics denote statistically significant differences compared to week 4 (in week 1 and 2).

The resting membrane potential (RMP) of neurons from both layers became more negative during the first postnatal week (Fig. 3b; L3, p < 0.001; L5, p < 0.001). A further hyperpolarising shift between weeks 2 and 4 did not reach significance. Interestingly, while input resistance - which has been found to correlate to size of the cell - also decreased during the second week, it decreased further after this (Fig. 3c; L3, p = 0.001; L5, p < 0.001). Simultaneously, the membrane time constant became faster during the first postnatal month (Fig. 3d; L3, p < 0.001; L5, p < 0.001).

Layer 5 neurons showed a characteristic voltage sag upon injection of hyperpolarising current, which is absent in layer 3 neurons (Fig. 3a). Layer 5 neurons showed a voltage sag that was larger at week 1 than at consecutive weeks (Fig. 3e; L5, p = 0.005). Thus, while actual values may differ between layers 3 and 5, the development of passive properties was similar for both layers.

Whereas several passive membrane properties continued to change through to week 4, properties of action potentials did not change after the second postnatal week. Action potential halfwidth (measured as the width of the action potential at the midpoint between threshold and peak) decreased substantially during this time in cells of both layers (Fig. 4a,b; L3, p < 0.001; L5, p < 0.001), with no further change occurring later. Similarly, action potential amplitude (Fig. 4c; L3, p < 0.001; L5, p < 0.001) increased, and action potential threshold (Fig. 4d; L3, p < 0.001; L5, p < 0.001) became more hyperpolarised up until week 2, with no further significant changes afterwards. In contrast, rheobase increased further until week 4, after an initial increase during the second postnatal week (Fig. 4e; L3, p < 0.001; L5, p < 0.001), reflecting the continued decrease in input resistance (Fig. 3c).

Cells in both layers displayed regular-spiking firing patterns. However, spike frequency adaptation (SFA) showed developmental changes that were distinct between layers. To quantify SFA, we measured the ratio between the 9^th^ interspike interval (ISI) and the 1^st^ (ISI 1/ISI 9) and the 4^th^ (ISI 4/ISI 9) as a measure of early and late adaptation, respectively. Cells in both layers showed a doublet in which the first spike of a train was followed rapidly by a second spike. Layer 3 neurons only developed this doublet during the second postnatal week (Fig. 4a). This was reflected by a significant decrease in the ISI 1/ISI 9 ratio between weeks 1 and 4 (Fig. 4f,g). Cells in layer 5, on the other hand, showed an initial doublet at all ages, but exhibited spike frequency accommodation at 1 week, which disappeared during the second postnatal week. (Fig. 4f,h; ISI 4/ISI 9, week 1 vs week 2, p < 0.001). In conclusion, the development of passive membrane properties, as well as properties of individual spikes, showed similar patterns for cells of layers 3 and 5, whereas responses to prolonged stimulation developed differently between layers.

### Differential development of synaptic input

**Figure 5:**
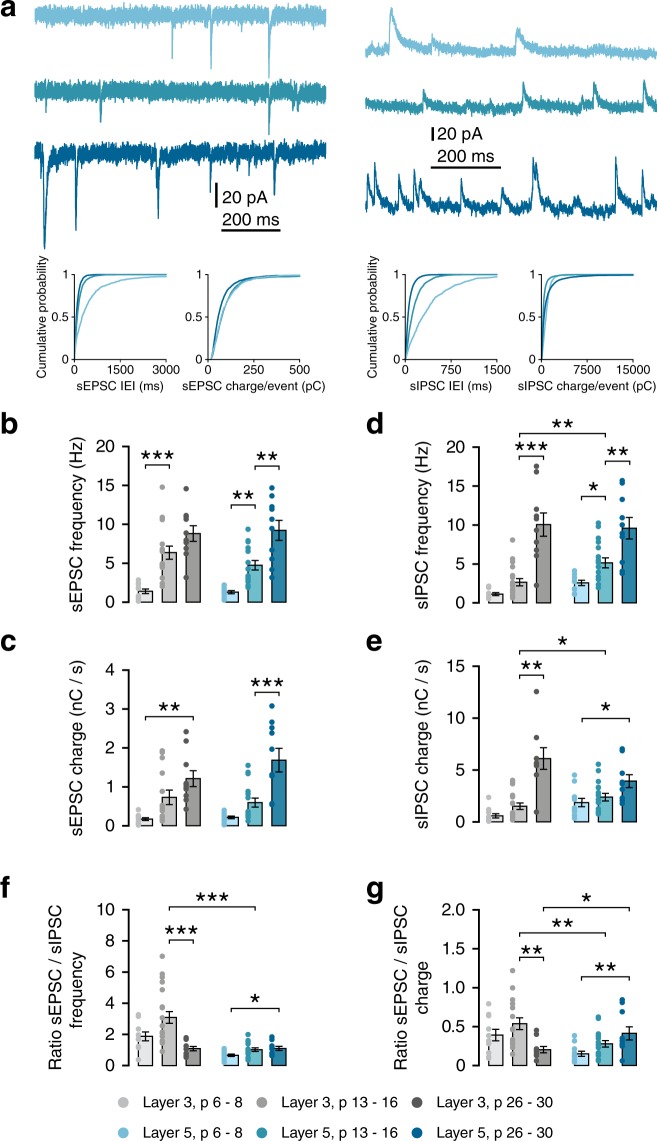
Development of spontaneous synaptic transmission follows lamina-specific patterns. (**a**) Example traces recorded at −70 mV (left) or +10 mV (right) from L5PNs at 1, 2 and 4 weeks. Bottom panels show probability distributions for inter-event-intervals and charge-per-event for single example cells. (**b**) sEPSC frequency increases in L3 during the second postnatal week (Welch’s F(2,17.8) = 36.1, p < 0.001; post-hoc w1–2, p < 0.001). sEPSCs frequency in L5 increases between weeks 1 and 4 (Welch’s F(2,13.3) = 30.13, p < 0.001; post-hoc w1–2, p = 0.006; w2–4, p = 0.001). (**c**) sEPSC charge/second increases from week 1 to 4 in L3 (Welch’s F(2,16.3) = 15.70, p = 0.002; w1-w4, p = 0.001) and from week 2 to 4 in L5 (Welch’s F(2,16.0) = 15.49, p < 0.001; w2–4, p < 0.001). (**d**) sIPSC frequency increases in L3 between weeks 2 and 4 (W’s F(2,17.0) = 23.53, p < 0.001; w2–4, p < 0.001) and until week 2 in L5 (F(2,34) = 15.58, p < 0.001; w1–2, p = 0.029; w2–4, p = 0.005). L3-L5 w2, t(31) = 3.19, p = 0.003. (**e**) sISPCs charge/second increases in week 2 to 4 in L3 (F(2,35) = 23.80, p < 0.001; w2–4, p < 0.001), and from week 1 to 4 in L5 (W’s F(2,17.9) = 18.22, p = 0.018; w1–2, p = 0.029; w1–4, p = 0.005). L3-L5 w2, M-W U = 76, p = 0.031. (**f**) Ratio of sEPSC and sIPSC frequencies measured in the same cells. In L3, E/I frequency decreases between weeks 2 and 4 (K-W H(3) = 13.57, p = 0.001; w2–4, p < 0.001). In L5, E/I frequency ratio increases between weeks 1 and 4 (F(2,34) = 1.60, p = 0.033; w1–4, p = 0.047). L3 vs L5: w2, Welch’s t(19.05) = 5.167, p < 0.001; w4, t(18) = 0.07, p = 0.948. (**g**) Ratio of sEPSC/sIPSC charge in L3 decreases between weeks 2 and 4 (K-W H(3) = 9.92, p = 0.007; w2–4, p = 0.005). In L5 cells, there is an increase between weeks 1 and 4 (F(2,34) = 1.64, p = 0.011; w1–4, p = 0.008). L3 vs L5: w2, Welch’s t(24.84) = 3.005, p = 0.006; w4, M-W U = 21, p = 0.029.

Since most aspects of dendritic morphology and intrinsic membrane properties developed in parallel in layers 3 and 5, we next asked whether synaptic input onto PNs in both layers also developed simultaneously. Further, we wondered whether the ratio of excitation and inhibition would show a similar developmental pattern. Hence, we assessed spontaneous excitatory (sEPSCs) and inhibitory (sIPSCs) postsynaptic currents in the same cells (Fig. 5a, Table 3). Interestingly, sEPSCs showed distinct patterns of development between layers. In layer 3 cells, sEPSC frequency plateaued after the second postnatal week, with no significant further increase up to week 4 (Fig. 5b, L3, w1–2, p < 0.001; w2–4, p = 0.099). sEPSC charge also showed the largest increase during the second postnatal week, although the change was only significant between weeks 1 and 4 (Fig. 5c). In contrast, sEPSC frequency and charge onto layer 5 cells increased only slightly during the second postnatal week, with a significant increase occurring between weeks 2 and 4 (Fig. 5b,c). sIPSCs showed an inverse pattern, with frequency increasing gradually in layer 5, and only after the second postnatal week in layer 3 (Fig. 5d). sIPSC charge showed the same development in layer 3 as did sIPSC frequency. In layer 5, sIPSC charge showed a small gradual increase between 1 and 4 weeks (Fig. 5e). Recording both sEPSCs and sIPSCs in the same cells allowed us to calculate E/I ratios per cell. At 2 weeks, synaptic input onto layer 3 cells was dominated by excitation, with L3 cells receiving over three times as many excitatory events as inhibitory ones (Fig. 5f; E/I frequency at w2, L3: 3.09 ± 0.38, L5: 1.02 ± 0.12). This resulted in an E/I ratio that was significantly higher at 2 weeks in layer 3 cells than layer 5 (Fig. 5g; E/I charge, w2, L3 vs L5, t(24.84) = 3.005, p = 0.006). Interestingly, the late increases in excitatory input onto layer 5 cells and inhibitory input onto layer 3 cells resulted in a switch at week 4, with E/I ratio being higher in layer 5 cells at that age (Fig. 5g; E/I charge, w4, L3 vs L5, M-W U = 21, p = 0.029).

**Table 3:** Properties of excitatory and inhibitory synaptic input onto layer 3 and 5 pyramidal neurons at 1, 2, and 4 weeks.

*Variable*	1 week p 7.2 ± 0.8 n = 11/8	2 weeks p 14.6 ± 1.0 n = 12/7	4 weeks p 28.2 ± 0.9 n = 10/7	*Omnibus test result*
**Layer 3**				
**sEPSC frequency (Hz)**	***1***.***39*** ± ***0***.29***	6.36 ± 0.84 (n = 17/10)	8.81 ± 1.00	W’s F(2,17.8) = 36.1 p < 0.001
**sEPSC charge (pC/s)**	*170*.*8* ± *35*.8**	732.1 ± 187.7 (n = 17/10)	1209.6 ± 204.7	W’s F(2,16.3) = 15.70 p = 0.002
**sEPSC amplitude (pA)**	**32**.**05** ± **2**.28*	23.53 ± 0.96	26.58 ± 1.37	W’s F(2,18) = 6.26 p = 0.009
**sEPSC rise time (ms)**	***0***.***56*** ± ***0***.02**	0.73 ± 0.05	0.79 ± 0.05	F(2,10) = 9.17 p = 0.005
**sEPSC decay time (ms)**	*2*.*97* ± *0*.21*	3.15 ± 0.13	3.71 ± 0.15	F(2,30) = 5.22 p = 0.011
**sIPSC frequency (Hz)**	*0*.*66* ± *0*.19***	*2*.*65* ± *0*.46***	9.59 ± 1.50	W’s F(2,17.0) = 23.53 p < 0.001
**sIPSC charge (pC/s)**	*574*.*2* ± *208*.3**	*1159*.*1* ± *316*.6 **	6098.4 ± 1,163.9	K-W H(3) = 22.27 p < 0.001
**sIPSC amplitude (pA)**	*42*.*38* ± *2*.96*	*40*.*13* ± *2*.54**	56.71 ± 5.05	H(2) = 8.30 p = 0.016
sIPSC rise time (ms)	1.20 ± 0.11	1.14 ± 0.10	1.48 ± 0.15	F(2,30) = 2.21 p = 0.128
**sIPSC decay time (ms)**	***17***.***71*** ± ***0***.70 ***	*13*.*57* ± *0*.51**	10.68 ± 0.36	W’s F(2,19) = 41.03 p < 0.001
**Layer 5**				
***Variable***	**1 week p 7**.**0** ± **0**.**8 n** = **11/9**	**2 weeks p 14**.**7** ± **0**.**9 n** = **11/7**	**4 weeks p 28**.**1** ± **1**.**1 n** = **10/8**	***Omnibus test result***
**sEPSC frequency (Hz)**	***1***.***28*** ± ***0***.20**	*4*.*75* ± *0*.06** (n = 16/10)	9.22 ± 1.29	W’s F(2,13.3) = 30.13 p < 0.001
**sEPSC charge (pC/s)**	***213***.***6*** ± ***33***.7***	*596*.*2* ± *115*.9*** (n = 16/10)	1683.8 ± 303.9	W’s F(2,16.0) = 15.49 p < 0.001
**sEPSC amplitude (pA)**	*29*.*36* ± *1*.82**	24.44 ± 1.37	34.49 ± 2.19	F(2,27) = 7.59 p = 0.002
sEPSC rise time (ms)	0.74 ± 0.08	0.84 ± 0.05	0.88 ± 0.07	H(2) = 3.57 p = 0.167
sEPSC decay time (ms)	3.88 ± 0.44	3.70 ± 0.24	3.61 ± 0.11	W’s F(2,15) = 0.22 p = 0.807
**sIPSC frequency (Hz)**	***2***.***10*** ± ***0***.34*	*5*.*14* ± *0*.64**	9.12 ± 1.38	W’s F(2,17.9) = 18.22 p < 0.001
**sIPSC charge (pC/s)**	*1859*.*4* ± *409*.1 *	2389.5 ± 371.1	3921.7 ± 650.1	F(2,33) = 0.46 p = 0.018
sIPSC amplitude (pA)	46.10 ± 2.75	40.13 ± 1.34	47.27 ± 2.58	F(2,27) = 2.63 p = 0.090
**sIPSC rise time (ms)**	*1*.*29* ± *0*.15*	*1*.*08* ± *0*.05*	0.85 ± 0.05	W’s F(2,17) = 6.85 p = 0.007
**sIPSC decay time (ms)**	***16***.***72*** ± ***1***.40**	10.21 ± 0.60	8.75 ± 0.71	W’s F(2,18) = 12.42 p < 0.001

Ages are given as mean ± sd in days postnatal. N given as number of cells/number of mice. Values are given as mean ± SEM. W’s F = Welch’s F. Asterisks denote post-hoc significance: *p < 0.05, **p < 0.01, ***p < 0.001. Bold values denote statistically significant differences compared to week 2 (in week 1), italics denote statistically significant differences compared to week 4 (in week 1 and 2).

**Figure 6:**
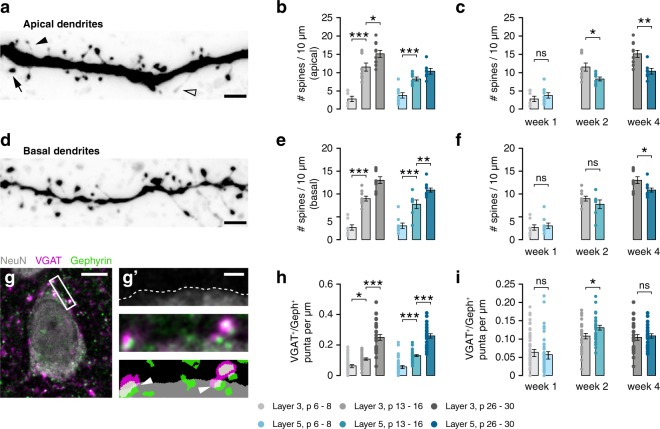
Dendritic spine densities show a similar developmental pattern across layers. (**a**) Example image of apical dendrite of a p14 L5 cell, showing mushroom (arrow), thin (closed arrowhead) and filopodium-like spines (open arrowhead). Scale bar 2 µm. (**b**) Development of apical spine densities (L3, F(2,23) = 37.37, p < 0.001; post-hoc w1-w2, p < 0.001, w2-w4, p = 0.034; L5, F(2,21) = 23.53, p < 0.001; post-hoc w1-w2, p < 0.001, w2-w4, ns). (**c**) Within-age-group comparisons of data in b. Apical spine density is higher in L3 neurons at both 2 and 4 weeks (w1, t(14) = 0.89, p = 0.387; w2, t(15) = 2.53, p = 0.023; w4, t(15) = 3.543, p = 0.003). (**d**) Example image of basal dendrite of a p14 L5 cell. Scale bar 2 µm. (**e**) Development of basal spine densities (L3, F(2,24) = 64.36, p < 0.001; post-hoc w1-w2, p < 0.001, w2-w4, p < 0.001; L5, F(2,24) = 42.95, p < 0.001; post-hoc w1-w2, p < 0.001, w2-w4, p = 0.006). (**f**) Within-age-group comparisons of data in e. Basal spine density is higher in L3 only at 4 weeks (w1, M-W U = 33, p = 0.815; w2, t(14) = 1.19, p = 0.255; w4, t(19) = 2.55, p = 0.020). (**g**) Quantification of perisomatic inhibitory synapses. Scale bar 5 µm. (**g’**) shows, from top to bottom, delineation of the soma, high magnification composite fluorescence image, and mask of thresholded image. Arrowheads indicate perisomatic synapses. Scale bar 1 µm. (**h**) The density of perisomatic inhibitory synapses increases during development in both layers (L3, H = 56.47, p < 0.001, post-hoc w1-w2, p = 0.022, w2-w4, p < 0.001; L5, H = 64.8, p < 0.001, post-hoc w1-w2, p < 0.001, w2-w4, p < 0.001). (**i**) The density of inhibitory synapses is higher in L5 neurons than L3 neurons at 2 weeks (w1, M-W U = 846, p = 0.638; w2, t(56) = 2.34, p = 0.023; w4, t(50) = 0.38, p = 0.708).

We next sought to see whether the differences in E/I ratio we found between layers have a structural correlate. To this end, we assessed dendritic spine densities on both apical and basal dendrites (Fig. 6a–f), as well as density of perisomatic inhibitory synapses (Fig. 6g–i). Similar to dendritic length, the density of dendritic spines increased most during the second week of development in both layers (Fig. 6b,e), as did the proportion of thick spines, which are more mature (Supplementary Fig. S2). After the first postnatal week, spine densities were not significantly different between layers (Fig. 6c,f). Overall spine density was higher in layer 3 cells at two weeks on apical dendrites but not basal dendrites (Fig. 6c,f). From week 2 to 4, the difference in spine densities between layers increased, with spine densities being higher in L3 neurons at 4 weeks on both apical and basal dendrites (Fig. 6c,f).

The density of perisomatic inhibitory synapses was assessed by immunohistochemical staining for the vesicular GABA transporter VGAT, the inhibitory postsynaptic protein gephyrin and the neuronal marker NeuN (Fig. 6g). The density of perisomatic inhibitory synapses increased drastically during the first postnatal month (Fig. 6h). At 2 weeks, we found a higher density of inhibitory synapses onto the soma of L5 neurons than on those in L3 (Fig. 6i). Therefore, at 2 weeks, densities of perisomatic inhibitory synapses, but not dendritic spines, were in line with physiologically measured laminar differences in synaptic input.

## Discussion

Because information is transferred through cortical layers in sequence^32^, knowledge of cross-layer neuronal development is necessary to understand early cortical circuit formation. For cortical regions that are essential for cognitive and executive functions, comprehending these processes is vital in order to advance our understanding of not only neurotypical development but also that of NDDs and neuropsychiatric conditions. The first postnatal month of rodent mPFC development is a period of significant change for formation, plasticity and maturation of excitatory synapses that is similar to other sensory neocortical regions, illustrated both by functional and structural alterations^33–35^. By combining functional and structural measurements of developing pyramidal neurons in mouse mPFC, we confirm the second postnatal week as a period of rapid growth, similar to that in other neocortical regions^16,36^. We show that while passive and active electrical properties develop similarly in deep and superficial neurons, the development of excitatory and inhibitory synaptic function differs, leading to distinct development of E/I balance between layers. These data underline the importance of layer-specific and developmental analyses for understanding cortical circuit formation and refinement.

Maturation of pyramidal neuron morphology in both layers is most pronounced during the first and second postnatal weeks in mouse mPFC, with overall dendrite length more than doubling from P6–8 to P13–16 for pyramidal neurons in both superficial and deep layers. Electrophysiologically, we find that action potentials become both larger in amplitude and faster during the second postnatal week. This is likely due to both maturation of ion channels^37,38^ and the observed changes in dendritic morphology, which impacts action potential dynamics^39^. Input resistance continues to decrease after week 2 in both layers, indicating an increase in leak current. This may be caused by an increase in surface area after the second postnatal week due to an increase in dendrite thickness, which we did not measure. Alternatively, the decrease in input resistance could be due to a decrease in specific membrane resistance, which would likely be mediated by members of the KCNK family of potassium leak channels^40^. For example, cortical expression of both TASK-3 and TWIK1 increases during postnatal development up to P28^41^. While input resistance shows the same developmental pattern in both layers, it remains to be determined whether the same channels mediate this change in both cell types. Other conductances show developmental profiles, depending on cell type. Cells in the later age group from both layers showed an initial doublet at the start of the spike train. Layer 3 PNs only develop an initial doublet after the second postnatal week, but do not otherwise show significant changes in SFA during development. Layer 5 PNs, on the other hand, show SFA during the latter half of the spike train at week 1, which disappears after the second postnatal week. The precise conductances underlying spike frequency adaptation are not fully understood^42–45^. Our results indicate that distinct ionic mechanisms underlie the initial doublet and later SFA, and that these mechanisms are regulated differentially across layers.

The hyperpolarisation-activated current I_H_, which is mediated by HCNs^46^, is largely absent in PNs in layer 3. While I_H_ increases during late development in layer 5 pyramidal neurons^47^, we find here that I_H_ is initially strong, and decreases substantially during the second postnatal week. In contrast, in pyramidal neurons in both hippocampal CA1 and CA3, H-currents increase in amplitude during early development^48^. Development of H-current in layer 5 cortical pyramidal neurons is thus distinct from that in hippocampal pyramidal neurons, and more resembles that in L1 interneurons^49^. By the end of the fourth postnatal week, morphological and intrinsic electrical properties of layer 5 pyramidal neurons such as RMP, input resistance and overall dendritic length are comparable to those reported for rat mPFC between P24–46^6^. Detailed developmental profiles for intrinsic properties of layer 5 rat mPFC pyramidal neurons from birth until adolescence/early adulthood suggest that many parameters, including input resistance and the membrane time constant, do not increase significantly after the third postnatal week into adulthood^18^.

Our data provide a detailed overview of the development of the dendritic morphology and intrinsic membrane properties of pyramidal neurons in layers 3 and 5 of the mouse mPFC. The PFC in humans is generally thought to develop later than hierarchically lower cortical areas, such as the primary visual cortex^50^. However, in rats, the prefrontal cortex does not seem to have a delayed development relative to other sensory cortical regions based upon neuronal morphology^18,51^ or excitatory synaptic transmission^52^. Our findings regarding dendritic growth in the mouse mPFC are similar to those of previous studies in other cortical areas of mouse and rat. For example, similar growth patterns for layer 2/3 pyramidal neurons occur in mouse S1^11^ and a detailed analysis of thick-tufted pyramidal neuron development in layer 5 of rat S1 found comparable neuronal maturation patterns to those in our data^16^. Thus, we find no evidence for developmentally-delayed maturation at a cellular level and conclude that dendritic morphology and intrinsic properties of mouse mPFC pyramidal neurons develop simultaneously to those of neurons in other cortical areas during this period. However, during adolescence, more subtle changes occur^16^. As we did not collect any data from animals older than four weeks, it is possible that these later subtle changes are different between mPFC and other cortical areas.

We show that in contrast to most cell-autonomous properties, synaptic excitatory and inhibitory innervation follow distinct developmental trajectories for neurons in either layer. Balance between excitation and inhibition is carefully maintained in the adult brain, with inhibition onto individual cells scaling with neuronal activity^53^. Interestingly, we find that E/I ratios vary across development and cortical layers. Especially at 2 weeks, the difference is striking: layer 5 neurons at this point receive approximately as many excitatory as inhibitory synaptic events, while layer 3 neurons receive relatively more excitation. This is reflected in a significantly higher E/I ratio in layer 3, in terms of both frequency and charge, showing distinct laminar regulation of E/I balance during development. It has been shown that GABA action is excitatory in early postnatal cortex^54^. However, at the end of the second postnatal week, GABA is inhibitory in slice preparations of somatosensory cortex^55^. It remains to be determined whether the GABA switch occurs simultaneously in the mPFC, and whether there are laminar differences in its timing. Interestingly, the difference in inhibitory inputs between layers is reflected in the density of perisomatic synapses these cells receive. In contrast, we did not see a similar correlation between spine densities and excitatory inputs, as L3 neurons show higher spine densities at both 2 and 4 weeks. However, this may be offset by their shorter dendritic length.

Our results contradict previous studies that find simultaneous and similar maturation of E/I balance across all layers of the somatosensory cortex^56^. There are several possible explanations for this discrepancy. First, Zhang *et al*. measured conductances in response to extracellular stimulation, whereas we measured spontaneous frequency. We therefore did not control the behaviour of the presynaptic cell(s). Second, this phenomenon may be specific to the mPFC. Measurement of excitatory and inhibitory responses to defined electrical or behavioural stimulation is needed to show whether this is the case.

Synapses from layer 3 to layer 5 in mPFC are still developing after 2 weeks^18^, whereas synapses onto layer 2/3 pyramidals develop faster^57^. Hence, the development of synapses, along with E/I balance, likely follows a complex pattern throughout the cortex. Our results support this view and indicate a layer-specific maturation of the balance of excitation and inhibition. Additionally, there are likely intralaminar differences in addition to translaminar ones. Corticofugal layer 5 neurons receive more inhibition than do layer 5 neurons that project intracortically^58,59^, and development of inhibitory synapses onto layer 5 pyramidal neurons in the cingulate cortex is dependent on the projection target of the postsynaptic cell^60^. It is therefore likely that developmental trajectories and timing of synapse formation also differ between cell types within a cortical layer.

The maturation of synaptic inhibition regulates critical time windows during development^61^. Critical (or sensitive) time windows refer to periods during development during which particular neuronal networks or synaptic pathways show heightened plasticity. The concept of sensitive time windows has also been applied to NDDs^22,62^. In this context, sensitive time windows represent transient periods during which networks are particularly vulnerable to synaptic dysfunction. Consistent with this, studies have found transient phenotypes in models for NDDs^25,26^. Such disruptions of network formation during these periods may lead to impairments later on in development^63^. Thus, the distinct synaptic maturation and development of E/I ratios between layers reported here may represent distinct sensitive time windows in cortical layers. We suggest that NDDs may not only show the transient age-related phenotypes reported previously, but that the occurrence and timing of these phenotypes may differ between cortical layers.

## Methods

### Slice preparation

All procedures involving animals were conducted in compliance with Dutch regulations and were approved by the animal experimental committee (“Dier ethische commissie (DEC)”; license number: INF 13-02) of the Vrije Universiteit. Animals were housed and bred according to institutional and Dutch governmental guidelines and regulations.

C57BL/6 males aged 6–8 days (1 week), 13–16 days (2 weeks) or 26–30 days (4 weeks) were rapidly decapitated and their brains dissected out in ice cold cutting solution containing (in mM): 110 choline chloride, 26 NaHCO_3_, 10 D-glucose, 11.6 sodium ascorbate, 7 MgCl_2_, 3.1 sodium pyruvate, 2.5 KCl, 1.25 NaH_2_PO_4_, and 0.5 CaCl_2_ (Bureau *et al*., 2006). 300 µm thick coronal slices containing the prelimbic cortex were obtained using a Microm HM 650 V vibratome (Thermo Scientific, Waltham, MA, USA), and allowed to recover at room temperature in aCSF containing (in mM): 125 NaCl, 26 NaHCO_3_, 10 D-glucose, 3 KCl, 2.5 MgSO_4_, 1.6 CaCl_2_, and 1.25 NaH_2_PO_4_, with an osmolality of ±300 mOSm, which was continuously bubbled with carbogen gas (95% O2, 5% CO_2_).

### Electrophysiology

Slices in the recording chamber were perfused with aCSF as described above, but with 1.5 mM MgSO_4_ and heated to 31 ± 1 °C. Pyramidal neurons in layers 3 and 5 were visualised using DIC on a BX51WI microscope with a 40 × /0.8 NA objective (Olympus, Tokyo, Japan) and IR camera (VX 45, PCO, Kelheim, Germany). Layer 3 pyramidal neurons were patched just below the dense band of layer 2 (150–225 µm from pia at week 1, 225–300 µm from pia at weeks 2 and 4; see Supplementary Fig. S1). Layer 5 neurons were identified by their larger soma size and location (300–420 µm from pia at week 1, 400–550 µm from pia at weeks 2 and 4). As mPFC lacks a layer 4, no cells were patched in the band between 225–300 µm (week 1) and 300–400 µm (weeks 2 and 4) to avoid cells whose layer identity might be ambiguous (Supplementary Fig. S1). Recordings were made using borosilicate (GC150–10, Harvard Apparatus, Holliston, MA) glass pipettes with a resistance of 3–5 MΩ, pulled on a horizontal puller (P-87, Sutter Instrument Co., Novato, CA). Signals were amplified (Multiclamp 700B, Molecular Devices) and digitised (Digidata 1440A, Molecular Devices) and recorded in pCLAMP 10 (Molecular Devices, Sunnyvale, CA). Series resistance was monitored before, during, and after recording. Cells were discarded if the series resistance deviated more than 25% from its value at the start of recording, or if it exceeded 20 MΩ.

To record membrane properties, pipettes were filled with an intracellular solution containing (in mM): 148 K-gluconate, 1KCl, 10 Hepes, 4 Mg-ATP, 4 K_2_-phosphocreatine, 0.4 GTP and 0.2% biocytin, adjusted with KOH to pH 7.3 ( ± 290 mOsm). During recording, a series of negative and positive current injections were applied. Active and passive properties were analysed in Matlab (Mathworks, Natick, MA) using custom scripts. The resting membrane potential was determined to be the membrane potential during the 0-mV current injection. Input resistance was calculated as the linear slope of the current-voltage (I-V) relationship of the last 200 ms of all negative stimuli. The membrane time constant was determined by fitting a single exponential to the first 300 ms of the response to the negative current injection that resulted in a voltage deflection of approximately 7.5 mV. Voltage sag was calculated as the percentage change between the peak of the response and the average voltage deflection of the last 200 ms of the same step.

Properties of individual action potentials were determined for the first current injection to elicit action potentials and averaged for all action potentials in that step. Action potential threshold was set as the voltage at which the first derivative of the voltage trace reached 20 V/s. Action potential amplitude was calculated as the difference between the threshold and the peak of each action potential. Distance to threshold was calculated as the difference between the average action potential threshold of a cell and its resting membrane potential. Interspike interval (ISI) ratios were determined for the first current injection to elicit 10 or more action potentials. Rheobase was determined by injecting a 5 s positive ramp current, the peak of which was adjusted according to the cell’s approximate input resistance.

To record spontaneous excitatory and inhibitory postsynaptic currents (sEPSCs/sIPSCs), pipettes were filled with an intracellular solution containing (in mM): 125 Cs-gluconate, 5 CsCl, 4 NaCl, 10 HEPES, 0.2 EGTA, 2 K_2_-phosphocreatine, 2 Mg-ATP, 0.3 GTP, adjusted with KOH to pH 7.3 (±290 mOsm).

sEPSCs and sIPSCs were recorded in the same cell. Recordings of 5 minutes were made per cell per synaptic event type. To record sEPSCs, cells were clamped at −70 mV. To record sIPSCs, cells were clamped at +10 mV. IPSCs were confirmed to be GABAergic by their abolishment by 10 µM Gabazine after several experiments. sEPSCs and sIPSCs were analysed using MiniAnalysis (SynaptoSoft, Decatur, GA, USA). Charge carried by sEPSCs and sIPSCs was determined as the total area of all events in a trace divided by the length of that trace. E/I ratios were calculated for each individual cell.

### Dendritic morphology and spines

Slices containing biocytin-filled cells were fixed in 4% paraformaldehyde in 1x PBS for 24–48 hrs at 4 °C. Slices were then washed at least 3 × 10 min in 1x PBS, and incubated in 1x PBS containing 0.5% Triton X-100 and 1:500 Alexa 488-streptavidin (Invitrogen, Waltham, MA) on a shaker at room temperature (RT) for 48 hrs. Slices were then washed at least 3 × 10 min in 1x PBS and mounted on glass slides in mowiol.

Confocal stacks were made of neurons that were evenly stained, oriented parallel to the slice surface and with no major dendrites cut. Neurons were imaged using an A1 confocal microscope (Nikon, Tokyo, Japan) using a 10x, NA 0.45 objective, scanned at 0.44 µm x 0.44 µm × 1.0 µm (xyz) resolution.

Cellular morphology was reconstructed using NeuroMantic software^64^. The resulting reconstructions will be submitted to NeuroMorpho.org. Reconstructions were quantitatively analysed using NeuronExplorer (MicroBrightfield Bioscience, Colchester, VT, USA). Dendritic segments were classified as intermediate, having a branch point at the distal end of the segment, or terminal, having no distal branch points. The length of the apical trunk was measured from the soma to the first bifurcation of the apical tuft.

Dendritic spines were imaged on the same microscope, using a 100x, NA 1.49 oil objective, scanned at 0.08 µm × 0.08 µm × 0.1 µm (xyz) resolution, and analysed using NeuronStudio^65^. Spines were classified based on their length, the presence and width of the spine head, according to the following scheme: Spines with length >3 µm and/or head diameter <0.3 µm were classified as filopodia. Stubby spines were defined as spines with a head diameter >0.3 µm and a length/head diameter ratio <1.5. Mushroom spines were defined as spines with head diameter between 0.3 µm and 0.6 µm and a length/head diameter between 1.5 and 3, or head diameter >0.6 µm and length/head diameter >1.5. Spines with head diameter between 0.3 µm and 0.6 µm and length/head diameter >3 were classified as thin. In our final analysis, stubby and mushroom spines were lumped together as thick spines, as non-super-resolution imaging techniques have been shown to overestimate the number of stubby spines^66^.

### Inhibitory synapse quantification

Inhibitory synapses were detected by immunohistochemical staining for vesicular GABA transporter (VGAT) and inhibitory postsynaptic protein gephyrin. Mice aged 7 days, 14 days, and 30 days were deeply anesthetized by intraperitoneal injection of lethal dose of sodium pentobarbitol, and transcardially perfused with saline, followed by 4% PFA in PBS. Brains were postfixed for 2 hours in 4% PFA, and then transferred in steps to 30% sucrose in PBS at 4 °C. 40 µm sections were cut using a sliding microtome (Leica Biosystems, Wetzlar, Germany). Free-floating sections were permeabilized in 0.25% Triton X-100 for 1 hour and then incubated in blocking solution containing 0.25% Triton X-100, 2% bovine serum albumin, 5% normal goat serum and 5% normal donkey serum in 1x PBS for 2 hours at room termperature. Primary antibodies were diluted in the same solution and incubated at 4 °C overnight. Sections were then washed in 1x PBS 4 × 10 minutes in 1x PBS. Sections were incubated with secondary antibodies in blocking solution for 2 hours at room temp, washed 4 × 10 minutes in 1x PBS, and mounted on glass slides in mowiol.

Antibodies used were: rabbit anti-NeuN (Millipore, ABN78), guinea pig anti-VGAT (Synaptic Systems, 131004), mouse IgG1 anti-gephyrin (Synaptic Systems, 147011), Goat anti-rabbit Alexa 405 (Abcam, ab175652), donkey anti-guinea pig Alexa 647 (Jackson, 706-605-148), goat anti-mouse IgG1 Alexa 555 (Invitrogen, A-21127).

Single focal plane images were taken using a Leica TSC-SP8 confocal, with 100 × 1.44 NA objective, and 2.2x digital zoom at 1024 by 1024 pixels (pixel size, 51.7 nm x 51.7 nm). The same laser power, gain and offset settings were used for all groups. Cells from both layers were imaged in each section. Afterwards, inhibitory synapses were detected in FIJI as areas of overlap between VGAT and gephyrin in thresholded images that were within 0.4 µm of the edge of the cell as identified by NeuN staining. Synapse densities are reported as number of synapses per µm of the perimeter of the cell.

### Statistics

All values are given as mean ± standard error of the mean (SEM). Statistical tests were performed using SPSS (IBM, Armonk, NY, USA) and Graphpad Prism 7 (Graphpad, San Diego, CA, USA). Omnibus tests were performed separately for both layers, as described below. False discovery rate was maintained at 5% using the procedure described by Benjamini and Hochberg^67^. This resulted in a p-value cut-off (α) of 0.036. Appropriate post-hoc tests were performed for omnibus tests that produced a significant p-value using α = 0.036.

For omnibus tests, residuals were checked for normality and homoscedasticity. For residuals that were normally distributed and homoscedastic, a one-way ANOVA was performed. The post-hoc test performed when the test produced a significant p-value was Tukey’s honest significance test. If variance was heteroscedastic, Welch’s correction was used, with a Games-Howell post-hoc test. If residuals were not normally distributed, but variance was homoscedastic, a Kruskal-Wallis test was performed, with Dunn’s test performed post-hoc. If residuals were not normally distributed and were not homoscedastic, a robust test was performed, based on 20% trimmed means using the WRS2 package in R. The post-hoc test used here was a percentile-bootstrapped multiple comparisons test using the *mcppb20* function. For count data, a generalised linear model was implemented using Poisson loglinear distribution. Estimated marginal means were calculated, using Šidák correction.

## Supplementary information

Supplementary files
