# Supplementary material for: Early postnatal development of pyramidal neurons across layers of the mouse medial prefrontal cortex
Source: Sci Rep. 2019 Mar 25;9:5037. doi: 10.1038/s41598-019-41661-9 (PMC6433913; doi:10.1038/s41598-019-41661-9)
Supplement: Supplementary file 1 — Supplementary files [file 41598_2019_41661_MOESM1_ESM.pdf]

Supplementary files

Early postnatal development of pyramidal neurons across layers of the mouse medial prefrontal cortex

Tim Kroon*[1,2], Eline van Hugte[1,3], Lola van Linge[1,4], Huibert D. Mansvelder[1], Rhiannon M. Meredith[1]

[1] Department of Integrative Neurophysiology, Center for Neurogenomics & Cognitive Research, Vrije Universiteit Amsterdam, De Boelelaan 1085, 1081 HV Amsterdam, The Netherlands.

[2] Present address: MRC Centre for Developmental Neurobiology, Institute of Psychiatry, Psychology & Neuroscience, King's College London, New Hunt's House, Guy's Campus, London SE1 1UL, UK

[3] Present address: Department Cognitive Neurosciences, Department of Human Genetics, Donders Institute for Brain, Cognition and Behaviour, Radboud University Medical Centre, Geert Grooteplein 10 Noord, 6500 HB Nijmegen, The Netherlands

[4] Present address: Department of Functional Genomics, Center for Neurogenomics & Cognitive Research, Vrije Universiteit Amsterdam, De Boelelaan 1085, 1081 HV Amsterdam, The Netherlands.

*To whom correspondence should be addressed.
Email: tim.kroon@kcl.ac.uk / tm.kroon@gmail.com
Tel: +44 (0) 207 848 6552

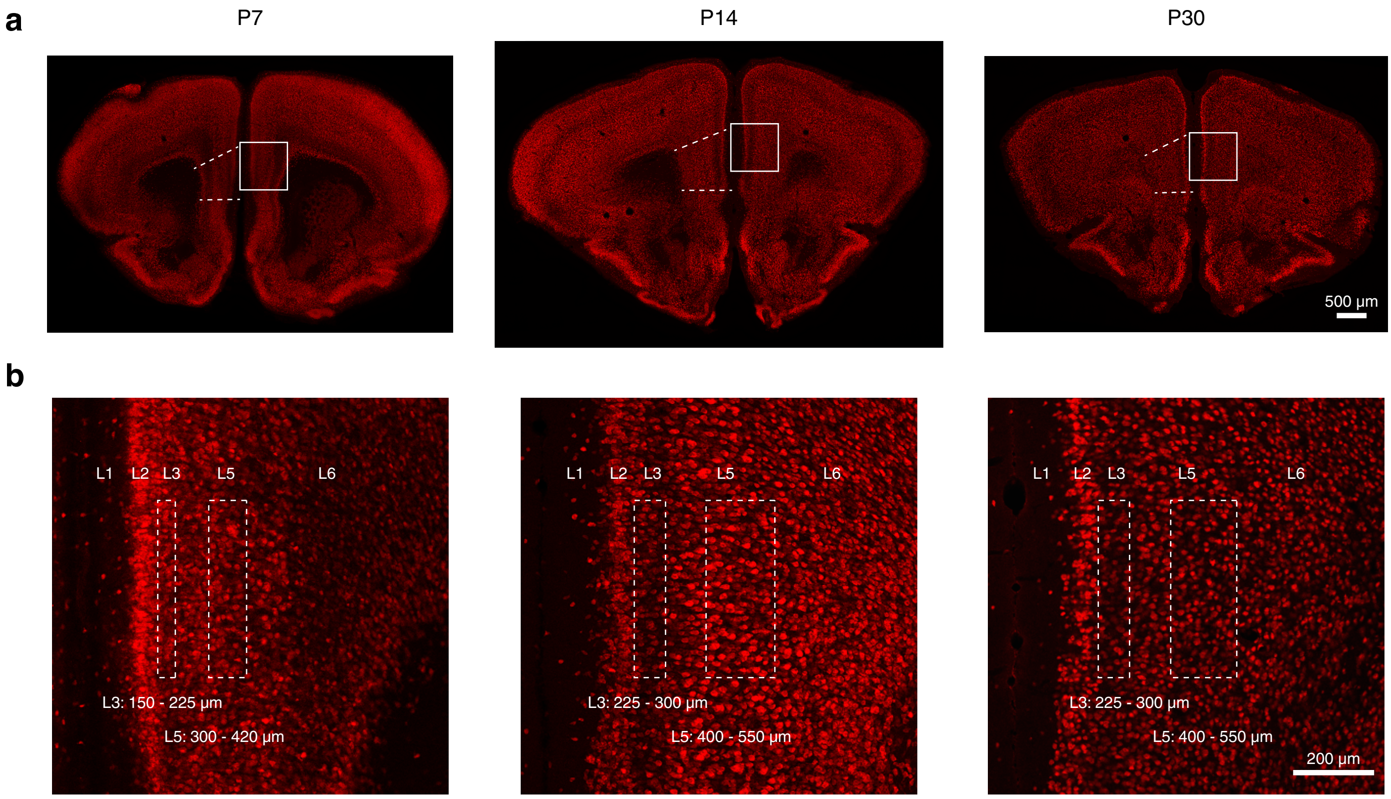

Figure S1. Targeting of mPFC pyramidal cells during development.

(a) Low magnification image of coronal sections from mice at the ages used in this study, stained for neuronal marker NeuN. Dashed lines indicate the prelimbic area where neurons were patched. Boxes indicate the location of the location of the higher magnification images shown in b.

(b) Confocal images of prelimbic cortex showing cortical layers. Boxes indicate the cortical depth at which neurons were targeted at these ages.

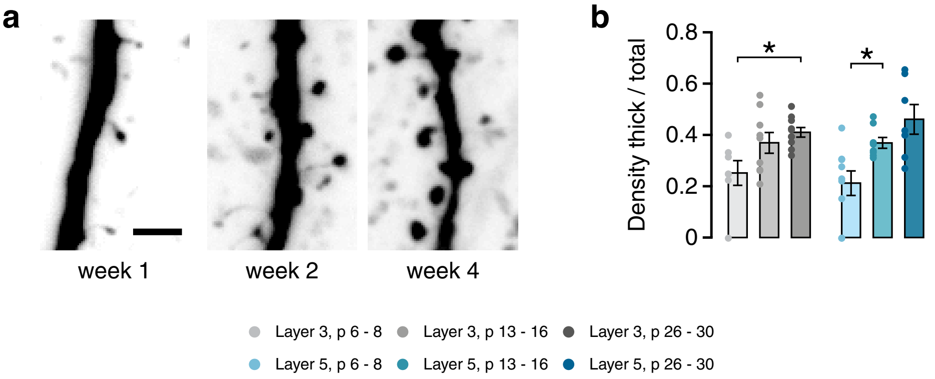

Figure S2. Developmental increase in the proportion of thick dendritic spines

(a) Example images showing dendritic spines on apical dendrites of L5 neurons. Scale bar 2 µm.
(b) The proportion of thick spines, as measured by (density of thick spines) / (total spine density), increases during development, (L3, $F(2,23) = 5.02$, $p = 0.016$; post-hoc w1-w4, $p = 0.013$; L5, $F(2,24) = 8.20$, $p = 0.002$; post-hoc w1-w2, $p = 0.044$, w2-w4, ns).
